# Effects of Apolipoprotein E polymorphism on carotid intima-media thickness, incident myocardial infarction and incident stroke

**DOI:** 10.1038/s41598-022-09129-5

**Published:** 2022-03-24

**Authors:** Anitha Pitchika, Marcello Ricardo Paulista Markus, Sabine Schipf, Alexander Teumer, Sandra Van der Auwera, Matthias Nauck, Marcus Dörr, Stephan Felix, Hans-Jörgen Grabe, Henry Völzke, Till Ittermann

**Affiliations:** 1 Institute for Community Medicine, University Medicine Greifswald, SHIP/Clinical-Epidemiological Research, Walther Rathenau Str. 48, 17475 Greifswald, Germany; 2 Department of Internal Medicine B, University Medicine Greifswald, Greifswald, Germany; 3 German Center for Cardiovascular Research (DZHK E.V.), Partner site Greifswald, Greifswald, Germany; 4 DZD (German Center for Diabetes Research), Site Greifswald, Greifswald, Germany; 5 Department of Medicine A, University Medicine Greifswald, Greifswald, Germany; 6 Department of Psychiatry and Psychotherapy, University Medicine Greifswald, Greifswald, Germany; 7 Institute of Clinical Chemistry and Laboratory Medicine, University Medicine Greifswald, Greifswald, Germany; 8 German Center for Neurodegenerative Diseases (DZNE), Site Rostock/Greifswald, Greifswald, Germany

**Keywords:** Cardiology, Risk factors

## Abstract

The Apolipoprotein E (APOE) gene polymorphism (rs429358 and rs7412) shows a well-established association with lipid profiles, but its effect on cardiovascular disease is still conflicting. Therefore, we examined the association of different APOE alleles with common carotid artery intima-media thickness (CCA-IMT), carotid plaques, incident myocardial infarction (MI) and stroke. We analyzed data from 3327 participants aged 20–79 years of the population-based Study of Health in Pomerania (SHIP) from Northeast Germany with a median follow-up time of 14.5 years. Linear, logistic, and Cox-regression models were used to assess the associations of the APOE polymorphism with CCA-IMT, carotid plaques, incident MI and stroke, respectively. In our study, the APOE E2 allele was associated with lower CCA-IMT at baseline compared to E3 homozygotes (β: − 0.02 [95% CI − 0.04, − 0.004]). Over the follow-up, 244 MI events and 218 stroke events were observed. APOE E2 and E4 allele were not associated with incident MI (E2 HR: 1.06 [95% CI 0.68, 1.66]; E4 HR: 1.03 [95% CI 0.73, 1.45]) and incident stroke (E2 HR: 0.79 [95% CI 0.48, 1.30]; E4 HR: 0.96 [95% CI 0.66, 1.38]) in any of the models adjusting for potential confounders. However, the positive association between CCA-IMT and incident MI was more pronounced in E2 carriers than E3 homozygotes. Thus, our study suggests that while APOE E2 allele may predispose individuals to lower CCA-IMT, E2 carriers may be more prone to MI than E3 homozygotes as the CCA-IMT increases. APOE E4 allele had no effect on CCA-IMT, plaques, MI or stroke.

## Introduction

Cardiovascular diseases (CVD) are the leading cause of death worldwide constituting 31% of all deaths^1^. Around 85% of these deaths are attributable to myocardial infarction (MI) and stroke. The etiology of CVD is multifactorial and the underlying genetic factors play a major role in the onset and development of CVDs by influencing quantitative traits such as blood pressure, adiposity or cholesterol levels^2^. The Apolipoprotein E (APOE) gene polymorphism is one of the most studied candidate genes for the risk of MI^3^, ischemic heart disease (IHD)^4^ and stroke^5^ apart from being investigated for type 2 diabetes^6^ and dementia^7^. APOE is a plasma protein consisting of 299 amino acids that plays an important role in cholesterol homeostasis and triglyceride metabolism by mediating the uptake of chylomicron remnants, very low-density lipoprotein, and intermediate density lipoprotein^8^. The polymorphic APOE gene is located on chromosome 19 and has three major alleles, E2, E3 and E4, resulting from the combination of 2 single nucleotide polymorphisms (SNP; rs429358 and rs7412) with amino acid interchanges of cysteine and arginine at residues 112 and 158, respectively.

The results of epidemiological studies with respect to MI and stroke are still conflicting. Some studies have reported the hazardous effects of the E4 allele in relation to MI^3,9^ and stroke^5,10^, while few others have shown the hazardous effects of the E2 allele^11,12^ and some even a protective effect of the E2 allele^3,13^. In addition, some studies found no association between APOE E2 or E4 alleles and MI^14–16^ and stroke^15,17^. Moreover, most of the studies on APOE gene are case–control in design, had smaller sample size (< 1000) and evidence from prospective population-based studies is still sparse. Indeed, three large population-based studies did not show any association between APOE and MI or stroke^15,18,19^.

Apart from CVD events, several studies exist on the association between APOE and carotid intima-media thickness (IMT) and carotid plaques^13,20–24^, which are non-invasive markers of arterial injury and atherosclerosis, respectively^25^. They have also been suggested to predict MI and stroke^26,27^, although carotid IMT is considered to be a weak predictor than carotid plaques^28,29^. The results pertaining to the effects of APOE alleles on carotid IMT and plaques have also been less clear and inconclusive. Some studies suggest that the E2 carriers had thinner carotid IMT and fewer plaques than E3 homozygotes but no association was seen in E4 carriers^13,20,21^, while others have reported that E4 carriers had increased carotid IMT but no association with respect to E2 carriers^22,23^ or carotid plaques^23^. A study by Beilby et al.^24^ found no association between E2 or E4 allele and carotid IMT but found E4 allele to be associated with increased risk of carotid plaque formation in men. A clear evidence of this association is vital, as it would further contribute to understand the role of APOE as a risk factor of CVD and the mechanism behind the relation.

Therefore, the aims of our study were (1) to investigate the association of the APOE allele with carotid IMT and plaques, and risk of incident MI and stroke during a median follow-up period of 14.5 years in a population-based cohort; (2) to investigate the influence of cholesterol levels on these associations and (3) to examine the effect of APOE alleles on the association between carotid artery measures of IMT and plaque at baseline and risk of incident MI and stroke.

## Materials and methods

### Study population

The Study of Health in Pomerania (SHIP) is a population-based project conducted in West Pomerania, Germany. For the first cohort, SHIP-START, the participants were recruited during 1997–2001 after a two-step stratified random sampling procedure based on age, sex and region. A total of 6265 subjects aged 20–79 years were drawn from the target population, of which 4308 participated in the baseline examination (response 68.8%). The follow-up examinations of the cohort were conducted during 1) 2002–2006 among 3300 participants (SHIP-START-1, response 83.6%); 2) 2008–2012 among 2333 participants (SHIP-START-2, response 67.4%) and 3) 2014–2016 among 1718 participants (SHIP-START-3, response 69.0%) after a mean follow-up duration from baseline of 5, 11 and 16 years, respectively. Details on the study design, protocols and sampling methods have been reported elsewhere^30,31^. All participants provided written informed consent. The study was approved by the medical ethics committee of the University of Greifswald and was conducted in accordance with the Helsinki Declaration.

### Interview and physical examination

All participants underwent a standardized computer-assisted personal interview, during which they provided information on sociodemographic and lifestyle factors as well as medical histories and medication use. The history of self-reported MI and stroke was collected by interview. During the first examination, the following questions were asked, (1) “Have you ever been diagnosed with heart attack by a doctor”, (2) “If yes, please specify the year of diagnosis of heart attack”, (3) “Have you ever been diagnosed with stroke by a doctor?”, (4) “If yes, please specify the year of diagnosis of stroke”. During the follow-up examinations, participants were asked similar questions whether they have been diagnosed with (1) MI or (2) stroke by a doctor since their last clinical visit and to mention the year of diagnosis. Furthermore, we linked data from health insurance records, mortality follow-up and a morbidity follow-up to our data. From these sources, MI and stroke were defined according to the ICD-10 code I21 and I63, respectively. Health insurance data were available quarterly from March 2002 upwards. Mortality follow-up data was available until March 2019 and we did consider all causes of death not only the main cause. The morbidity follow-up was conducted in 2006/2007. We included both nonfatal and fatal myocardial infarction and stroke in our study.

Information on smoking status was categorized into current, former and never smoking and alcohol consumption into no (0 g/day), moderate (men 0.1–39.9 g/day and women 0.1–19.9 g/day), and high alcohol (men ≥ 40 g/day and women ≥ 20 g/day) consumption. Participants who exercised for less than an h/week in their leisure time during summer or winter were classified as preferring a sedentary lifestyle. Participants were asked to bring all medications taken 7 days before the time of examination. Medication data were obtained online using the IDOM software (online drug database led medication assessment) and categorized according to the Anatomical Therapeutical Chemical (ATC) classification index. Antidiabetic medication was defined by the ATC code A10, antihypertensive medication by the ATC code C02, C03, C07, C08 and C09.

During the physical examination, standardized measurements of height, weight, and blood pressure were performed. Body mass index (BMI) was calculated. Blood pressure was measured three times on the right arm in a sitting position after at least 5-min at rest, using an oscillometric device (OMRON HEM 705-CP, Osaka, Japan). Systolic and diastolic blood pressures were calculated as the average reading of the second and third measurements. Participants were classified as hypertensive based on blood pressure readings ≥ 140/90 mmHg or use of antihypertensive medication. Participants were classified as diabetic based on self-reported physician diagnosis or use of antidiabetic medication (ATC code A10) or glycated hemoglobin (HbA1c) > 6.5%.

### Measurement of laboratory parameters

Non-fasting blood samples were taken and serum samples were analyzed within 1 h or stored at – 80^ °^C. Total, high-density lipoprotein (HDL) and low-density lipoprotein (LDL) cholesterol were measured photometrically (Hitachi 704, Roche, Mannheim, Germany). Serum triglycerides were determined enzymatically using reagents from Roche Diagnostics (Hitachi 717; Roche, Mannheim, Germany). High-sensitive C-reactive protein (hs-CRP) was determined immunologically on a Behring Nephelometer II with commercially available reagents (Dade Behring, Eschborn, Germany). HbA1c measured in whole blood was determined by high-performance liquid chromatography (Bio-Rad Diamat, Munich, Germany).

### Carotid ultrasound

Common carotid artery IMT (CCA-IMT) of the extracranial carotid arteries was examined bilaterally with B-mode ultrasound using a 5 MHz transducer and a high-resolution instrument (Diasonics VST Gateway, Santa Clara, USA) in participants over 45 years of age. Plaques were defined as any focal thickening of the intima-media complex protruding into the vessel lumen or as a focal increase of echogenicity with a homogeneously hyper-echoic echo texture within an otherwise hypo-echoic intima-media complex. The presence of plaques was assessed in the CCA, the bifurcations as well as in the internal and external carotid arteries. Certified readers calculated the mean far-wall CCA-IMT by averaging the 10 consecutive measurement points (in 1 mm steps) from the bulb of both sides, measuring in plaque-free areas only. Mean CCA-IMT was calculated as follows: (right mean IMT + left mean IMT)/2. Further information on the ultrasound protocol has been described elsewhere^32^.

### APOE genotyping

Participants were successfully genotyped using the Genome-Wide Human SNP Array 6.0 (Affymetrix, Santa Clara, California). The total number of SNPs identified after imputation and quality control was 2,748,910. Genotypes were determined using the Birdseed2 clustering algorithm. The genetic data analysis workflow was created using the Software InforSense. Imputation of genotypes was performed using the HRCv1.1 reference panel and the Eagle and minimac3 software implemented in the Michigan Imputation Server for pre-phasing and imputation, respectively. SNPs with a Hardy Weinberg Equilibrium p-value < 0.0001, a call rate < 0.95, and a minor allele frequency < 1% were removed before imputation. The APOE genotypes were determined on the basis of rs429358 (C; T) and rs7412 (T; C) from the resulting imputation (overall imputation quality > 0.8; Hardy Weinberg Equilibrium, p > 0.05)^33^. As we used the data from the genome-wide SNP chip instead of strand-specific genotyped SNPs for determination of APOE status, two ambiguous SNP combinations occurred where APOE E2/E4 and E1/E3 could not be discriminated (http://www.snpedia.com/index.php/APOE). The participants with the E2/E4 genotype were excluded as they possess a combination of potentially protective and risk alleles.

### Statistical analysis

For the analyses of APOE polymorphism, participants were classified as (1) E4 carriers who had at least one E4 allele (E3/E4 and E4/E4 genotypes), (2) E2 carriers who had at least one E2 allele (E2/E3 and E2/E2 genotypes), and E3 homozygotes (E3/E3). Baseline characteristics of the study participants were expressed as median and interquartile range for continuous data and as absolute numbers and percentages for categorical data. Differences in characteristics between E3 homozygotes, E2 carriers and E4 carriers were tested by Kruskal Wallis test for continuous data and χ^2^ test for categorical data. First, linear regression models were performed to investigate the association of APOE alleles with CCA-IMT measured at baseline. Second, multinomial logistic regression models were performed to investigate the association of APOE alleles with presence of 1–3 carotid plaques and ≥ 4 carotid plaques compared to no plaques. Third, Cox-proportional hazards regression models were performed to test the association of APOE alleles with incident MI and stroke, separately. We excluded subjects with previous MI or stroke at baseline from this analysis. For calculating the time of occurrence of MI or stroke obtained from different sources, we first defined the time point for MI or stroke onset in each source separately. For the study visits, we used the self-reported year of diagnosis of the event. The date of diagnosis was assumed to be the latest possible value (31st December) of the year mentioned by the subjects. In subjects who had not reported the year of diagnosis, the time of occurrence of MI or stroke was assumed to be the midpoint at which it was first reported and the previous visit. For the health insurance data we took the middle of the quarter, when MI or stroke was reported the first time; for the mortality follow-up we took the day of death; and for the morbidity follow-up we took the day of data entry. The day of MI and stroke diagnosis was defined as the earliest date reported in any of the above listed sources, and the follow-up time was calculated as time between the first study visit and the day of MI and stroke diagnosis. For individuals not developing the event, the censor date was the last study visit of the participant. Associations were analyzed based on stepwise adjustment for covariates at baseline for all four outcomes. The first model was adjusted for age and sex; the second model was further adjusted for BMI, smoking status, alcohol consumption, sedentary lifestyle, presence of hypertension and diabetes; and the third additionally adjusted for LDL and HDL cholesterol. Further, Cox-proportional hazards regression models were performed to investigate the association of CCA-IMT and carotid plaques measured at baseline with incident MI and stroke. The first model and second model were adjusted as mentioned previously. The third model was further adjusted for APOE allele status to check its influence on the association and the fourth model was additionally adjusted for LDL and HDL cholesterol. Further, we analyzed the effect of interactions between APOE alleles and CCA-IMT or carotid plaques on incident MI and stroke. For sensitivity analyses, we performed (1) subgroup analyses according to age (> 60 and < 60) and sex to check for associations with APOE polymorphism in specific subgroups; (2) competing risk regression models to examine the associations of APOE alleles on incident MI and stroke with all-cause mortality as the competing event; (3) longitudinal analysis to test the association of APOE polymorphism with MI and stroke observed at baseline as well as follow-up through logistic mixed effects models with time varying covariates and accounting for repeated observations within individuals; (4) Cox-regression models to examine the association of APOE alleles with a composite endpoint combining MI and stroke; (5) Cox- regression models to check the association of age adjusted IMT quintiles with incident MI and stroke and (6) logistic regression models to check the association of APOE alleles with carotid plaque prevalence (yes vs. no). Age adjusted IMT quintiles were calculated based on age categories < 50, 50–59.9, 60–69.9 and ≥ 70. At each age group, we categorized the IMT values into 5 groups based on percentile cut-offs at 20, 40, 60 and 80, following the study by Tosetto et al.^34^. A p value < 0.05 was considered as statistically significant. All analyses were carried out using Stata 13.1 (Stata Corporation, College Station, TX, USA).

## Results

Our study sample size varied according to specific outcome and analysis, which included a maximum of 3327 individuals out of 4308 individuals examined at baseline. The study participants had a median follow-up of 14.5 years (range 4.7–18.2 years). We excluded participants who were lost to follow-up (n = 755), who had missing APOE genotype data (n = 76), who carried the APOE E2/E4 genotype (n = 53), and those with previous MI (n = 146), previous stroke (n = 98) and further missing MI (n = 52) and stroke (n = 64) data alone. For the analysis of CCA-IMT, we included 2257 participants > 45 years of age and with CCA-IMT measurements at baseline.

In our study sample, the prevalence of APOE E3 homozygotes, E2 carriers and E4 carriers were 64.7%, 14.6% and 20.8%, respectively. At baseline, the lipid profile, CCA-IMT and hs-CRP levels differed significantly between the APOE allele groups. E4 carriers exhibited more often dyslipidemia, and thereby higher LDL and total cholesterol but lower HDL cholesterol and hs-CRP levels, whereas E2 carriers exhibited lower LDL, total cholesterol levels, and CCA-IMT but higher hs-CRP and triglyceride levels than E3 homozygotes (Table 1). There was no significant difference in BMI, sedentary lifestyle, type 2 diabetes, hypertension and previous MI or stroke at baseline between the APOE allele groups (Table 1).

**Table 1:** Characteristics of the study population at baseline stratified by APOE carrier status (n = 3327).

Variables	E3 homozygotes n = 2152	E2 carriers n = 485	E4 carriers n = 690	p value*
Age (years)	49 (36–61)	48 (34–60)	49 (37–60)	0.21
Sex, males	1051 (48.84%)	220 (45.36%)	328 (47.54%)	0.37
BMI (kg/m^2^)	26.7 (23.7–29.9)	27.1 (24.1–30.1)	27.1 (23.9–30.1)	0.34
**Smoking status**				
Non-smoker	789 (36.75%)	174 (36.10%)	257 (37.30%)	0.72
Ex-smoker	732 (34.09%)	178 (36.93%)	229 (33.24%)	
Curent smoker	626 (29.16%)	130 (26.97%)	203 (29.46%)	
**Alcohol consumption**				
No intake	308 (15.38%)	61 (13.53%)	93 (14.29%)	0.56
Moderate intake	1511 (75.47%)	356 (78.94%)	504 (77.42%)	
High intake	183 (9.14%)	34 (7.54%)	54 (8.29%)	
Sedentary lifestyle	1214 (56.57%)	259 (53.73%)	391 (56.75%)	0.50
LDL cholesterol (mmol/l)	**3.26 (2.68–3.87)**	**2.90 (2.44–3.54)**	**3.37 (2.73–4.01)**	**0.0001**
HDL cholesterol (mmol/l)	1.26 (1.01–1.53)	1.24 (1.02–1.53)	1.20 (0.98–1.48)	0.08
Total cholesterol (mmol/l)	**5.4 (4.7–6.1)**	**5.0 (4.4–5.7)**	**5.4 (4.7–6.2)**	**0.0001**
Triglycerides (mmol/l)	**1.5 (1.06–2.23)**	**1.64 (1.12–2.42)**	**1.59 (1.09–2.36)**	**0.045**
Hs-CRP (mg/l)	**1.28 (0.66–2.93)**	**1.32 (0.70–2.67)**	**1.10 (0.53–2.74)**	**0.006**
Carotid intima-media thickness	**0.76 (0.68–0.86)**	**0.74 (0.64–0.85)**	**0.76 (0.67–0.87)**	**0.03**
**Carotid plaques**				
0	393 (32.86%)	87 (34.80%)	125 (33.24%)	0.32
1–3	538 (44.98%)	121 (48.40%)	162 (43.09%)	
≥ 4	265 (22.16%)	42 (16.80%)	89 (23.67%)	
Hypercholesterolemia	**896 (45.69%)**	**164 (37.88%)**	**337 (53.32%)**	**0.0001**
Obesity	528 (24.56%)	122 (25.26%)	181 (26.23%)	0.67
Diagnosed with type 2 diabetes	211 (9.85%)	52 (10.81%)	57 (8.35%)	0.34
Previous MI	97 (3.77%)	21 (3.68%)	22 (2.65%)	0.31
Incident MI	155 (7.43%)	34 (7.26%)	55 (8.17%)	0.79
Previous stroke	50 (1.94%)	13 (2.28%)	22 (2.63%)	0.47
Incident stroke	147 (6.97%)	22 (4.63%)	49 (7.24%)	0.15
Diagnosed with hypertension	1134 (52.72%)	255 (52.69%)	378 (53.14%)	0.63

In the regression analysis of APOE and CCA-IMT at baseline, we found that E2 carriers had lower mean CCA-IMT (β: − 0.02 [95% CI − 0.04, − 0.004]) than E3 homozygotes when adjusting for age, sex, BMI, smoking status, alcohol consumption, sedentary lifestyle, hypertension and type 2 diabetes (Table 2). The inverse association of E2 allele with CCA-IMT slightly attenuated and was no longer significant after adjustment for LDL and HDL cholesterol (β: − 0.01 [95% CI − 0.03, 0.01]). We found no significant association between E4 allele and CCA-IMT in any of the models (β: 0.01 [95% CI − 0.01, 0.02]). In our stratified analyses, the inverse effects between E2 allele and CCA-IMT were stronger and significant in men (β: − 0.03 [95% CI − 0.06, − 0.004]) while not in women (β: − 0.01 [95% CI − 0.04, 0.01]). Further, in our study, 899 (44.79%) had 1–3 carotid plaques and 499 (24.86%) had ≥ 4 carotid plaques. We observed that E2 carriers had lower odds of ≥ 4 carotid plaques (OR: 0.56 [95% CI 0.36, 0.87]) than E3 homozygotes. The inverse association slightly attenuated after adjustment for LDL and HDL cholesterol but remained statistically significant (OR: 0.61 [95% CI 0.38, 0.999]). E2 carriers also demonstrated a trend towards negative association with carotid plaque prevalence (OR: 0.73 [95% CI 0.53, 1.01]) as a dichotomous outcome. E4 carriers showed no association with carotid plaques.

**Table 2:** Association of APOE allele status (E2 and E4 carriers versus E3 homozygotes) with carotid intima thickness, carotid plaques, incident myocardial infarction and stroke.

Outcome	Model	N	E2 carriers Estimate (95% CI)	p	E4 carriers Estimate (95% CI)	p
***β * coefficients**						
Carotid intima-media thickness*	1	2257	**− 0.02 (− 0.04, − 0.01)**	**0.01**	0.01 (− 0.01, 0.02)	0.31
	2	2025	**− 0.02 (− 0.04, − 0.004)**	**0.02**	0.004 (− 0.01, 0.02)	0.58
	3	1783	− 0.01 (− 0.03, 0.01)	0.19	0.003 (− 0.01, 0.02)	0.75
**Odds ratios**						
Number of plaques 1–3 plaques vs. no plaques	1	899/1508	0.85 (0.61, 1.17)	0.32	0.94 (0.71, 1.25)	0.68
	2	896/1499	0.81 (0.58, 1.12)	0.20	0.93 (0.70, 1.23)	0.61
	3	794/1331	0.80 (0.55, 1.15)	0.23	0.89 (0.66, 1.21)	0.46
Number of plaques ≥ 4 plaques vs. no plaques	1	499/1108	**0.61 (0.40, 0.93)**	**0.02**	1.09 (0.77, 1.53)	0.63
	2	499/1102	**0.56 (0.36, 0.87)**	**0.01**	1.13 (0.79, 1.61)	0.52
	3	427/964	**0.61 (0.38, 0.999)**	**0.05**	1.14 (0.78, 1.68)	0.50
**Hazard ratios**						
Incident myocardial infarction	1	244/3226	1.07 (0.74,1.56)	0.71	1.03 (0.76, 1.40)	0.86
	2	226/2993	0.93 (0.62, 1.39)	0.71	0.99 (0.72, 1.37)	0.97
	3	200/2711	1.06 (0.68, 1.66)	0.78	1.03 (0.73, 1.45)	0.88
Incident stroke	1	218/3262	0.70 (0.44, 1.09)	0.11	0.93 (0.67, 1.30)	0.69
	2	199/3024	0.69 (0.43, 1.11)	0.12	0.85 (0.59, 1.20)	0.35
	3	177/2738	0.79 (0.48, 1.30)	0.35	0.96 (0.66, 1.38)	0.81

Significant associations are shown in bold font.

Model 1 adjusted for age and sex; Model 2 adjusted for model 1 + baseline values of body mass index, smoking status, alcohol consumption, sedentary lifestyle, hypertension and type 2 diabetes; Model 3: model 2 + low-density lipoprotein and high-density lipoprotein cholesterol.

*Carotid intima-media thickness and plaques were assessed only at baseline.

During the follow-up period, 244 MI events and 218 stroke events were reported in a follow-up time of 38,217 person-years and 38,684 person-years, respectively. The associations between APOE gene polymorphism and risks of incident MI and stroke are shown in Table 2. When compared with E3 homozygotes, both APOE E2 and APOE E4 carriers showed no significant association with incident MI (E2 HR: 1.07 [95% CI 0.74, 1.56]; E4 HR: 1.03 [95% CI 0.76, 1.40]) and incident stroke (E2 HR: 0.70 [95% CI 0.44, 1.09]; E4 HR: 0.93 [95% CI 0.67, 1.30]) even in the age- and sex-adjusted model. The association of APOE E2 and E4 carriers with incident MI (E2 HR: 1.06 [95% CI 0.68, 1.66]; E4 HR: 1.03 [95% CI 0.73, 1.45]) and incident stroke (E2 HR: 0.79 [95% CI 0.48, 1.30]; E4 HR: 0.96 [95% CI 0.66, 1.38]) did not change considerably after adjusting for smoking status, alcohol consumption, sedentary lifestyle, hypertension, type 2 diabetes, LDL and HDL cholesterol. Further, E2 and E4 carriers showed no significant associations with incident MI and stroke in our subgroup analyses of participants older than 60 years of age, younger than 60 years of age, males and females (data not shown). We found no association of APOE alleles with incident MI and stroke even in the competing risk model with all-cause death as the competing event (Supplementary Table 1). In the longitudinal analysis of MI and stroke including cases reported at baseline as well as follow-up, a total of 357 MI and 280 stroke events were observed in a total sample of 3904 and 3906 subjects, respectively. E2 and E4 carriers showed no associations with MI (E2 OR: 0.58 [95% CI 0.15, 2.25]; E4 OR: 0.85 [0.27, 2.68]) and stroke (E2 OR: 0.63 [95% CI 0.25, 1.60]; E4 OR: 1.24 [0.58, 2.62]) even in the longitudinal analysis (Supplementary Table 2). Further, APOE alleles were not associated with the composite endpoint of MI and stroke (Supplementary Table 3).

In our study, a standard deviation (SD) increase in CCA-IMT at baseline was associated with increased risk of incident MI (HR: 1.24 [95% CI 1.07, 1.45]) and stroke (HR: 1.39 [95% CI 1.19, 1.62], Table 3). The HR for incident MI slightly attenuated after adjustment for LDL and HDL cholesterol, but the HR for incident stroke remained almost similar (Table 3). Further, when compared to the lowest quintile, the highest age adjusted IMT quintile was associated with higher risk of incident stroke but not with incident MI (Supplementary Table 4). Further, presence of 1–3 carotid plaques at baseline was associated with higher risk of incident stroke (HR: 1.57 [95% CI 1.02, 1.41]), while presence of ≥ 4 carotid plaques at baseline was associated with a higher risk of both incident MI (HR: 1.68 [95% CI 1.02, 2.75]) and stroke (HR: 1.96 [95% CI 1.17, 3.26], Table 3). The associations remained similar after adjustment for APOE allele status but slightly attenuated after adjustment for LDL and HDL cholesterol. In the effect modification analyses of CCA-IMT by APOE allele status, E2 carriers showed a pronounced risk for incident MI (HR: 1.77 [95% CI 1.13, 2.76]) than E3 homozygotes (HR: 1.09 [95% CI 0.87, 1.37]) per SD increase in CCA-IMT at baseline (p value of interaction = 0.05, Table 4). There was no significant interaction between APOE alleles and carotid plaques on the association with MI and stroke.

**Table 3:** Association between carotid measures of intima-media thickness and plaques at baseline and risk of incident myocardial infarction and stroke.

Exposure	Model	Incident MI HR (95% CI)	p	Incident stroke HR (95% CI)	p
CCA-IMT per SD increase	1	**1.29 (1.10, 1.50)**	**0.001**	**1.46 (1.25, 1.69)**	** < 0.0001**
	2	**1.24 (1.07, 1.45)**	**0.006**	**1.39 (1.19, 1.62)**	** < 0.0001**
	3	**1.25 (1.07, 1.46)**	**0.005**	**1.37 (1.17, 1.60)**	**0.0001**
	4	1.14 (0.94, 1.36)	0.18	**1.38 (1.17, 1.63)**	**0.0001**
Carotid plaques 1–3 plaques vs. no plaques	1	1.24 (0.82, 1.87)	0.31	**1.59 (1.04, 2.44)**	**0.03**
	2	1.28 (0.83, 1.96)	0.26	**1.57 (1.02, 2.41)**	**0.04**
	3	1.28 (0.83, 1.96)	0.26	**1.56 (1.02, 2.40)**	**0.04**
	4	1.20 (0.76, 1.92)	0.43	1.40 (0.89, 2.21)	0.15
Carotid plaques ≥ 4 plaques vs. no plaques	1	**1.85 (1.16, 2.95)**	**0.01**	**2.19 (1.33, 3.60)**	**0.002**
	2	**1.68 (1.02, 2.75)**	**0.04**	**1.96 (1.17, 3.26)**	**0.01**
	3	**1.67 (1.01, 2.75)**	**0.04**	**1.97 (1.18, 3.28)**	**0.01**
	4	1.62 (0.93, 2.82)	0.09	1.63 (0.93, 2.85)	0.09

Significant associations are shown in bold font. Model 1 adjusted for age and sex; Model 2 adjusted for model 1 + baseline values of body mass index, smoking status, alcohol consumption, sedentary lifestyle, hypertension and type 2 diabetes; Model 3: model 2 + APOE allele status; Model 4: model 3 + low-density lipoprotein and high-density lipoprotein cholesterol.

*CCA-IMT* common carotid artery intima-media thickness, *SD* standard deviation.

**Table 4:** Modification of associations between carotid artery measures of intima-media thickness and plaque at baseline and risk of incident MI and stroke by APOE allele status.

	E3 homozygotes	E2 carriers	E4 carriers	p int E2 vs. E3	p int E4 vs. E3
**Hazard ratios for incident MI**					
CCA-IMT per SD increase	1.09 (0.87, 1.37)	**1.77 (1.13, 2.76)**	0.99 (0.68, 1.43)	**0.05**	0.63
Carotid plaques 1–3 vs. no plaques	0.99 (0.56, 1.74)	3.01 (0.66, 13.71)	1.24 (0.51, 3.03)	0.17	0.66
Carotid plaques 4–8 vs. no plaques	**1.96 (1.05, 3.68)**	2.12 (0.41, 11.01)	0.90 (0.33, 2.48)	0.93	0.17
**Hazard ratios for incident stroke**					
CCA-IMT per SD increase	**1.38 (1.14, 1.66)**	1.61 (0.88, 2.94)	1.33 (0.94, 1.88)	0.62	0.86
Carotid plaques 1–3 vs. no plaques	1.29 (0.76, 2.21)	3.55 (0.44, 28.81)	1.40 (0.56, 3.49)	0.36	0.89
Carotid plaques 4–8 vs. no plaques	1.55 (0.80, 3.02)	2.79 (0.30, 25.94)	1.78 (0.68, 4.64)	0.62	0.81

Significant associations are shown in bold font. Model adjusted for age, sex, baseline values of body mass index, smoking status, alcohol consumption, sedentary lifestyle, hypertension and type 2 diabetes, low-density lipoprotein and high-density lipoprotein cholesterol.

*p int* p value of interaction, *CCA-IMT* common carotid artery intima-media thickness, *SD* standard deviation.

## Discussion

In this population-based cohort study, we observed that the APOE E2 carriers had lower mean CCA-IMT and lower odds of ≥ 4 carotid plaques than E3 homozygotes, while we found no significant association of E2 alleles with incident MI and incident stroke. Further, CCA-IMT and presence of carotid plaques ≥ 4 at baseline were associated with increased risk of incident MI and stroke. However, the association between CCA-IMT and increased risk of incident MI was more pronounced in E2 carriers than E3 homozygotes, although E2 carriers had lower mean CCA-IMT in general. APOE E4 allele had no effect on CCA-IMT, carotid plaques, incident MI or stroke.

In general, E4 allele is considered as a potential risk allele whereas E2 is considered as a protective allele. In our study, we found some evidence to support E2 being protective with respect to lower CCA-IMT and lower odds of carotid plaques than E3 homozygotes, similar to previous studies^13,20,21,35^. A recent multiethnic exome-wide association study including 25,109 participants found that APOE E2 carriers had lower CCA-IMT than non-carriers^20^. A study conducted in 189 Finnish men reported lower CCA-IMT in E3/E2 carriers^35^. The Rotterdam population-based study including 5401 participants^21^ and a meta-analysis of five studies including 11,641 participants^13^ have both shown a mean difference of 0.02 mm lower CCA-IMT in E2 carriers than in E3 homozygotes. This estimate is similar to our study, probably due to the fact that all three studies included people of European ancestry. There was no significant difference in CCA-IMT between E4 carriers and E3 homozygotes in all four studies. However, some studies have reported that E4 carriers had higher CCA-IMT in the whole population^23^, in diabetic men^36^ and in patients with IHD^37^, while another study found no effect of APOE E2 or E4 allele on CCA-IMT^24^, in contrast to our study. A meta-analysis observed higher CCA-IMT in E4 carriers when compared to E2 carriers^22^, which is consistent with our study (data not shown). While the results are inconsistent, the majority of the studies support the presence of lower CCA-IMT in E2 carriers than E3 homozygotes. Studies examining the association between APOE alleles and carotid plaques are scarce. The inverse association between APOE E2 alleles and carotid plaques have also been observed in the Rotterdam study^21^, which is in line with our study. However, two other studies have reported no association^23^ or increased risk of carotid plaques in E4 carriers^24^, respectively.

Dyslipidemia is known to be a risk factor for atherosclerosis and accordingly, studies have shown associations between cholesterol levels and CCA-IMT^38^ and carotid plaques^39^. Further, favorable changes in lipids have shown to reduce the progression of CCA-IMT^40^. In addition, it is well established that APOE E2 carriers have lower cholesterol levels and E4 carriers have higher cholesterol levels than E3 homozygotes^41,42^. Since the APOE gene is related to cholesterol metabolism as well as CCA-IMT and carotid plaques, we investigated the influence of cholesterol levels on the association between APOE and CCA-IMT and plaques. We observed that the inverse association slightly attenuated after adjustment for LDL and HDL cholesterol. Previous studies have used slightly different adjustments for cholesterol levels. A study conducted in Finnish men reported an attenuation of negative effect on CCA-IMT in E3/E2 genotype after adjusting for LDL and HDL cholesterol^35^, similar to our study. A population-based study from Rotterdam observed slight attenuation of effects on CCA-IMT but not carotid plaques after adjustment for total and HDL cholesterol^21^. As a sensitivity analysis, we used different adjustments for cholesterol such as total, LDL and HDL cholesterol separately and in combinations. In all the models, the effect of E2 allele on CCA-IMT and carotid plaques slightly attenuated (data not shown). Overall, this implies that the lower CCA-IMT and lower risk for carotid plaques in E2 carriers might be partly due to their lowering effect on cholesterol levels.

Several case–control studies have shown associations between APOE E4 allele and IHD in populations including participants with MI, angina pectoris, coronary stenosis, coronary angioplasty or coronary artery bypass surgery^4,41,43,44^. A recent study from the UK Biobank including 391,992 participants reported that E3/E4 and E4/E4 genotype had higher risk of IHD, while E2/E3 genotype had lower risk of IHD when compared to E3/E3 genotype^41^. Our study included a narrow endpoint of MI alone and did not show any association with APOE polymorphism, in accordance with some studies^14–16^, but contrary to other ones^3,9^. Moreover, a large population-based study from Rotterdam including 6852 participants found no association between APOE polymorphism and MI or stroke^15^, which is consistent with our study. Similarly, a large population-based study from Utrecht including 7418 participants also found no association between APOE polymorphism and IHD and ischemic stroke^45^. In addition, several other prospective population-based studies have also reported no association between APOE E4 allele and stroke risk^15,18,19,46,47^, similar to our study. In contrast, two meta-analyses based on case–control studies suggest that APOE E4 allele may be associated with an increased risk of stroke^10,48^. The discrepancies in findings may possibly be due to differences in study design, sample size and study population.

APOE E2 allele was not significantly associated with MI or stroke, which is consistent with results from a meta-analysis and few studies^5,10,15,16,19^. On the other hand, recent meta-analyses have suggested a protective effect of E2 allele on MI^3,9^ and stroke^13^. Our study observed negative HR estimates for the association between E2 allele and stroke risk, although not significant. It is possible that our study might have lacked sufficient statistical power to detect a protective effect of E2 allele.

Some studies have suggested age- and sex- specific effects of APOE polymorphism on IHD and stroke^4,18^. A study from the UK Biobank suggested that E2/E2 genotype may have a protective effect for IHD in older population (> 60 years of age) while it might increase the risk for IHD in younger population^4^. A study from the Iowa cohort reported that E2 carriers had lower risk of stroke in subjects < 80 years of age, but the protective effect was not seen in the older population^18^. However, in our study we observed no age-specific protective or harmful effects of APOE polymorphism on MI or stroke. Thus, we have no consistent evidence for age-specific effects in our study as well as in the literature.

We further examined associations of CCA-IMT and carotid plaques with incident MI and stroke. We also performed several sensitivity analyses such as analyzing age adjusted IMT quintiles and carotid plaque prevalence on the outcomes. In all the analyses, CCA-IMT and carotid plaques were associated with increased risk of incident stroke. On the other hand, CCA-IMT and presence of ≥ 4 carotid plaques showed significant associations with incident MI but not age adjusted IMT quintiles or carotid plaque prevalence. Further, the associations could be partially explained by cholesterol levels, except the association between CCA-IMT and incident stroke which remained similar after adjustment for cholesterol levels. We can observe that the association between carotid measures and incident stroke are stronger than with incident MI.

In our study, APOE E2 allele was associated with lower CCA-IMT and lower risk of carotid plaques than E3 homozygotes. We also found CCA-IMT and carotid plaques to be associated with increased risk of incident MI and stroke. However, we found no associations between APOE alleles and incident MI and stroke. Hence, we further explored whether the associations of CCA-IMT and carotid plaques with risk of incident MI and stroke were modified by APOE allele status. We observed that, when compared to E3 homozygotes, E2 carriers showed a pronounced risk for incident MI per SD increase in CCA-IMT at baseline in the model adjusted for all potential confounders including cholesterol levels. Hence, although E2 carriers had lower mean CCA-IMT than E3 homozygotes, their risk of incident MI is higher than E3 homozygotes as the CCA-IMT increases. There was no significant interaction between APOE alleles and carotid plaques on the association with MI and stroke. Further studies exploring the interaction between APOE alleles and carotid artery measures on incident MI and stroke are warranted.

Our study has limitations. First, APOE was determined by using imputed genotype data from SNP array and not by a specific assay for APOE. However, the overall imputation quality score was > 0.8. Second, the individuals with E2/E2 (n = 19) and E4/ E4 (n = 33) genotype were limited, thus we examined the association based on APOE alleles and not individual genotypes. Third, the outcomes MI and stroke were partly based on self-reported history of a doctor-diagnosis but the participants were asked a wide range of questions related to the outcomes at every follow-up, and their responses were checked thoroughly for any inconsistencies. Furthermore, we had access to health insurance records, mortality and morbidity follow-up records to identify additional cases, even though they were not available for all participants. Thus, some degree of non-differential misclassification of MI or stroke cannot be excluded. We do not expect it to be a major concern since the APOE alleles might not have an influence on the availability of the records, and the misclassification, if exists, might be regardless of the APOE E2, E3 or E4 alleles. Fourth, our study specifically examines the relationship between APOE and MI, but a broader endpoint like IHD, which is not available in our study, may have shown associations with APOE similar to other studies. Fifth, CCA-IMT and carotid plaques were only measured at baseline. Thus, only a cross-sectional but not longitudinal analysis was performed. Major strengths of our study include a large prospective population-based sample with a maximum follow-up time of nearly 18 years and the availability of various covariates to account for potential confounding.

Overall, our findings from a large population-based follow-up study suggest that APOE E2 allele is associated with lower mean CCA-IMT and lower odds of carotid plaques compared to E3 homozygotes. This might be partially explained by lower cholesterol levels in E2 carriers. However, neither APOE E2 allele nor E4 allele was associated with incident MI or incident stroke. Our findings are consistent with the large population-based study from Rotterdam, where E2 allele was associated with lower CCA-IMT^21^ but there was no association of APOE alleles with MI or stroke^15^. Our study also adds further evidence to the association between carotid artery measures of IMT and carotid plaques and risk of incident MI and stroke. However, the association between CCA-IMT and increased risk of incident MI was more pronounced in E2 carriers than E3 homozygotes. This implies that while APOE E2 allele may predispose individuals to lower CCA-IMT, E2 carriers may be more prone to MI than E3 homozygotes as the CCA-IMT increases. The association between CCA-IMT and incident stroke was not modified by APOE allele status. Further, APOE E4 allele, which is generally considered to be a risk allele, had no effect on CCA-IMT, carotid plaques, MI and stroke. Further large population-based studies exploring the influence of APOE alleles on the association between carotid artery measures and MI or stroke are warranted.

## Supplementary Information

Supplementary Information.

## Data Availability

The data that support the findings of the study are available on reasonable request from the corresponding author. The data are not publicly available due to privacy or ethical restrictions.

## Abbreviations

APOE: Apolipoprotein E
ATC: Anatomical therapeutical chemical
CVD: Cardiovascular disease
CCA-IMT: Common carotid artery intima-media thickness
HDL: High density lipoprotein
hs-CRP: High sensitive C-reactive protein
IHD: Ischemic heart disease
IMT: Intima-media thickness
LDL: Low-density lipoprotein
MI: Myocardial infarction
SHIP: Study of health in Pomerania
SNP: Single nucleotide polymorphism
SD: Standard deviation

## Code availability

The code generated during the study is available upon reasonable request from the corresponding author.
