# Supplementary material for: Effects of Apolipoprotein E polymorphism on carotid intima-media thickness, incident myocardial infarction and incident stroke
Source: Sci Rep. 2022 Mar 24;12:5142. doi: 10.1038/s41598-022-09129-5 (PMC8948289; doi:10.1038/s41598-022-09129-5)
Supplement: Supplementary file 1 — Supplementary Information. [file 41598_2022_9129_MOESM1_ESM.docx]

**Effects of Apolipoprotein E polymorphism on carotid intima-media thickness, incident myocardial infarction and incident stroke**

Anitha Pitchika^1^, Marcello Ricardo Paulista Markus^2,3,4^, Sabine Schipf^1,5^, Alexander Teumer^1,3^, Sandra Van der Auwera^6,8^, Matthias Nauck^3,7^, Marcus Dörr^2,3^, Stephan Felix^2,3^, Hans-Jörgen Grabe^6,8^, Henry Völzke^1,3,4^, Till Ittermann^1^

^1^ Institute for Community Medicine, University Medicine Greifswald, Greifswald, Germany

^2^ Department of Internal Medicine B, University Medicine Greifswald, Greifswald, Germany

^3^ German Center for Cardiovascular Research (DZHK e.V.), Partner site Greifswald, Greifswald, Germany

^4^ DZD (German Center for Diabetes Research), Site Greifswald, Greifswald, Germany

^5^ Department of Medicine A, University Medicine Greifswald, Greifswald, Germany

^6^ Department of Psychiatry and Psychotherapy, University Medicine Greifswald, Greifswald, Germany

^7^ Institute of Clinical Chemistry and Laboratory Medicine, University Medicine Greifswald, Greifswald, Germany

^8^ German Center for Neurodegenerative Diseases (DZNE), Site Rostock/Greifswald, Germany

**Corresponding author:**

Anitha Pitchika

University of Medicine Greifswald,

Institute for Community Medicine, SHIP/ Clinical-Epidemiological Research,

Walther Rathenau Str. 48,

17475 Greifswald, Germany

Phone +49 3834 86 7552

Email [anitha.pitchika@uni-greifswald.de](mailto:anitha.pitchika@uni-greifswald.de)

**Supplementary Table 1:** Competing risk model of the association of APOE E2 and E4 carriers versus E3/E3 genotype with incident myocardial infarction and stroke after considering all-cause death as the competing event

| **Outcome** | **Model** | **N** | **E2 carriers**  **Subhazard ratio (95% CI)** | **P** |  | **E4 carriers**  **Subhazard ratio (95% CI)** | **p** |
| --- | --- | --- | --- | --- | --- | --- | --- |
| *Competing risk regression with all-cause death as the competing event* | | | | | | | |
| Incident myocardial infarction | 1 | 3226 | 1.11 (0.77, 1.62) | 0.57 |  | 1.15 (0.85, 1.56) | 0.37 |
|  | 2 | 2993 | 0.97 (0.65, 1.45) | 0.88 |  | 1.08 (0.78, 1.48) | 0.65 |
|  | 3 | 2711 | 1.09 (0.70, 1.69) | 0.70 |  | 0.98 (0.70, 1.38) | 0.91 |
| Incident stroke | 1 | 3262 | 0.70 (0.44, 1.09) | 0.11 |  | 1.09 (0.78, 1.51) | 0.61 |
|  | 2 | 3024 | 0.70 (0.43, 1.12) | 0.14 |  | 1.06 (0.75, 1.49) | 0.75 |
|  | 3 | 2738 | 0.77 (0.47, 1.28) | 0.32 |  | 1.05 (0.73, 1.51) | 0.80 |
| Model 1 adjusted for age and sex; Model 2 adjusted for model 1 + baseline values of body mass index, smoking status, alcohol consumption, sedentary lifestyle, hypertension and type 2 diabetes; Model 3: model 2 + low-density lipoprotein and high-density lipoprotein cholesterol | | | | | | | |

**Supplementary Table 2:** Longitudinal association of APOE E2 and E4 carriers versus E3/E3 genotype with myocardial infarction and stroke

| **Outcome** | **Model** | **N (N obs)** | **E2 carriers**  **OR (95% CI)** | **P** |  | **E4 carriers**  **OR (95% CI)** | **p** |
| --- | --- | --- | --- | --- | --- | --- | --- |
|  | | | | | | | |
| Myocardial infarction | 1 | 3,913 (10,753) | 1.04 (0.43, 2.50) | 0.94 |  | 0.86 (0.40, 1.85) | 0.69 |
|  | 2 | 3,904 (10,676) | 0.58 (0.15, 2.25) | 0.43 |  | 0.85 (0.27, 2.68) | 0.79 |
|  | 3 | 3,833 (9,935) | 0.62 (0.14, 2.86) | 0.54 |  | 0.71 (0.20, 2.55) | 0.60 |
| Stroke | 1 | 3,916 (10,748) | 0.66 (0.26, 1.67) | 0.38 |  | 1.17 (0.55, 2.47) | 0.69 |
|  | 2 | 3,907 (10,669) | 0.63 (0.25, 1.60) | 0.33 |  | 1.24 (0.58, 2.62) | 0.58 |
|  | 3 | 3,836 (9,933) | 0.65 (0.26, 1.61) | 0.35 |  | 1.15 (0.55, 2.43) | 0.71 |
| N, Number of participants; N obs, Number of observations. Logistic mixed effects model was performed. Model 1 adjusted for age and sex; Model 2 adjusted for model 1 + baseline values of body mass index, smoking status, alcohol consumption, sedentary lifestyle, hypertension and type 2 diabetes; Model 3: model 2 + low-density lipoprotein and high-density lipoprotein cholesterol | | | | | | | |

**Supplementary Table 3:** Association of APOE E2 and E4 carriers versus E3/E3 genotype with a composite endpoint of myocardial infarction (MI) and stroke

| **Outcome** | **Model** | **N** | **E2 carriers**  **HR (95% CI)** | **P** |  | **E4 carriers**  **HR (95% CI)** | **p** |
| --- | --- | --- | --- | --- | --- | --- | --- |
| Composite MI and stroke | 1 | 3217 | 0.93 (0.69, 1.25) | 0.63 |  | 0.99 (0.78, 1.24) | 0.90 |
|  | 2 | 2987 | 0.87 (0.63, 1.19) | 0.38 |  | 0.94 (0.73, 1.20) | 0.60 |
|  | 3 | 2705 | 0.94 (0.67, 1.32) | 0.72 |  | 0.99 (0.76, 1.28) | 0.93 |
| Model 1 adjusted for age and sex; Model 2 adjusted for model 1 + baseline values of body mass index, smoking status, alcohol consumption, sedentary lifestyle, hypertension and type 2 diabetes; Model 3: model 2 + low-density lipoprotein and high-density lipoprotein cholesterol | | | | | | | |

**Supplementary Table 4:** Association between age adjusted intima-media thickness quintiles and risk of incident MI and stroke

| **Model** | **N** | **Incident MI**  **HR (95% CI)** | **p** |  | **Incident stroke**  **HR (95% CI)** | **p** |
| --- | --- | --- | --- | --- | --- | --- |
| *Age adjusted carotid intima-media thickness quintiles* | | | | | | |
| 1 | 1^st^ quintile | 1.00 (Ref) |  |  | 1.00 (Ref) |  |
|  | 2^nd^ quintile | 1.12 (0.66, 1.90) | 0.68 |  | 1.49 (0.86, 2.57) | 0.16 |
|  | 3^rd^ quintile | 1.60 (0.97, 2.66) | 0.07 |  | **2.00 (1.16, 3.45)** | **0.01** |
|  | 4^th^ quintile | 1.46 (0.87, 2.45) | 0.15 |  | 1.66 (0.95, 2.91) | 0.08 |
|  | 5^th^ quintile | 1.62 (0.97, 2.71) | 0.07 |  | **2.91 (1.71, 4.94)** | **0.0001** |
| 2 | 1^st^ quintile | 1.00 (Ref) |  |  | 1.00 (Ref) |  |
|  | 2^nd^ quintile | 1.09 (0.63, 1.90) | 0.75 |  | 1.40 (0.81, 2.44) | 0.23 |
|  | 3^rd^ quintile | 1.55 (0.91, 2.62) | 0.11 |  | **1.89 (1.09, 3.28)** | **0.02** |
|  | 4^th^ quintile | 1.47 (0.86, 2.52) | 0.16 |  | 1.47 (0.83, 2.59) | 0.19 |
|  | 5^th^ quintile | 1.53 (0.89, 2.62) | 0.12 |  | **2.59 (1.50, 4.45)** | **0.001** |
| 3 | 1^st^ quintile | 1.00 (Ref) |  |  | 1.00 (Ref) |  |
|  | 2^nd^ quintile | 1.08 (0.62, 1.88) | 0.78 |  | 1.37 (0.79, 2.38) | 0.26 |
|  | 3^rd^ quintile | 1.55 (0.91, 2.63) | 0.11 |  | **1.83 (1.06, 3.16)** | **0.03** |
|  | 4^th^ quintile | 1.47 (0.86, 2.53) | 0.16 |  | 1.38 (0.78, 2.44) | 0.27 |
|  | 5^th^ quintile | 1.53 (0.89, 2.63) | 0.12 |  | **2.49 (1.45, 4.28)** | **0.001** |
| 4 | 1^st^ quintile | 1.00 (Ref) |  |  | 1.00 (Ref) |  |
|  | 2^nd^ quintile | 1.05 (0.59, 1.86) | 0.87 |  | 1.33 (0.73, 2.41) | 0.36 |
|  | 3^rd^ quintile | 1.34 (0.77, 2.33) | 0.30 |  | 1.74 (0.97, 3.15) | 0.07 |
|  | 4^th^ quintile | 1.20 (0.68, 2.12) | 0.53 |  | 1.60 (0.88, 2.94) | 0.13 |
|  | 5^th^ quintile | 1.09 (0.61, 1.96) | 0.77 |  | **2.50 (1.38, 4.51)** | **0.002** |
| Significant associations are shown in bold font. Model 1 adjusted for age and sex; Model 2 adjusted for model 1 + baseline values of body mass index, smoking status, alcohol consumption, sedentary lifestyle, hypertension, and type 2 diabetes; Model 3: model 2 + APOE allele status; Model 3: model 2 + low-density lipoprotein and high-density lipoprotein cholesterol | | | | | | |
